# Obesity-Related Metabolic Risk in Sedentary Hispanic Adolescent Girls with Normal BMI

**DOI:** 10.3390/children5060079

**Published:** 2018-06-15

**Authors:** Gert-Jan van der Heijden, Zhiyue J. Wang, Zili D. Chu, Morey Haymond, Pieter J. J. Sauer, Agneta L. Sunehag

**Affiliations:** 1 Department of Pediatrics, Baylor College of Medicine, Houston, TX 77030, USA; mhaymond@bcm.edu (M.H.); asunehag@gmail.com (A.L.S.); 2 Department of Radiology, University of Texas Southwestern Medical Center, Dallas, TX 75390, USA; JERRY.WANG@childrens.com; 3 Department of Radiology, Baylor College of Medicine, Houston, TX 77030, USA; zchu@bcm.edu; 4 Department of Pediatric Radiology, Texas Children’s Hospital, Houston, TX 77030, USA; 5 Department of Pediatrics, Beatrix Children’s Hospital, University Medical Center Groningen, University of Groningen, Groningen 9713 GZ, The Netherlands; saupie46@gmail.com

**Keywords:** BMI, body fat, abdominal fat, Hispanic, insulin resistance

## Abstract

Hispanic adolescent girls with normal BMI frequently have high body fat %. Without knowledge of body fat content and distribution, their risk for metabolic complications is unknown. We measured metabolic risk indicators and abdominal fat distribution in post-pubertal Hispanic adolescent girls with Normal BMI (N-BMI: BMI < 85th percentile) and compared these indicators between girls with Normal BMI and High Fat content (N-BMI-HF: body fat ≥ 27%; *n* = 15) and Normal BMI and Normal Fat content (N-BMI-NF: body fat < 27%; *n* = 8). Plasma concentrations of glucose, insulin, adiponectin, leptin and Hs-CRP were determined. Insulin resistance was calculated using an oral glucose tolerance test. Body fat % was measured by DXA and subcutaneous, visceral and hepatic fat by MRI/MRS. The N-BMI-HF girls had increased abdominal and hepatic fat content and increased insulin resistance, plasma leptin and Hs-CRP concentrations (*p* < 0.05) as compared to their N-BMI-NF counterparts. In N-BMI girls, insulin resistance, plasma insulin and leptin correlated with BMI and body fat % (*p* < 0.05). This research confirms the necessity of the development of BMI and body fat % cut-off criteria per sex, age and racial/ethnic group based on metabolic risk factors to optimize the effectiveness of metabolic risk screening procedures.

## 1. Introduction

Obesity in adolescence is related to well-known metabolic derangements such as insulin resistance, high blood pressure, dyslipidemia, type 2 diabetes and the development of cardiovascular disease in adulthood [1]. The Body Mass Index (BMI) (weight in kg/(height in m)^2^) [2] is used to distinguish between individuals with a normal and high risk of developing obesity-related complications. BMI, however, is not an optimal marker of leanness/obesity [3], and adolescents with normal BMI can have high fat mass [3,4,5]. Fat content [3,4,5,6,7] and the specific pattern of high visceral and low subcutaneous abdominal fat distribution, ectopic fat deposition and adipose tissue dysfunction contribute to obesity-related complications [8,9,10,11,12,13]. Low grade chronic whole body inflammation as a result of oxidative and endoplasmic reticulum stress related to nutrient excess is related to cardiovascular disease (CVD) in adults and is elevated in obese adolescents [14,15,16]. In addition, it is proposed that adipocyte dysfunction is related to obesity-related morbidity [14,17,18]. Leptin resistance is related to CVD risk [14], whereas a high adiponectin concentration is purported to have a protective effect on insulin sensitivity partly attributed to its anti-inflammatory function [14,18]. It is currently not known if whole body inflammation and adipocyte dysfunction are present in adolescents with normal BMI.

At present, the potential discrepancy between BMI and obesity-related metabolic risk is recognized in specific racial/ethnic groups, predominately in the Asian population [19,20,21].

In addition, it has been known that the Hispanic population is at a high risk of obesity and obesity-related illnesses [22,23,24,25]. Hispanic adolescent girls have a relatively high body fat percentage in comparison with their white and black counterparts [5,26]. Moreover, in screening procedures for previous studies [8,16,27], we noted that 60% of sedentary Hispanic adolescent girls with a normal BMI have a high body fat % (≥27%) [28]. It is currently not known if a normal BMI in sedentary Hispanic adolescents with a normal or high body fat % is an indicator of the absence of metabolic risk, especially in girls with a high body fat %.

To address this issue, a wide range of metabolic risk indicators and body fat distributions using state of the art body composition measurements were measured in sedentary normal BMI Hispanic girls.

## 2. Materials and Methods

### 2.1. Participants

After approval of the protocol by the Baylor College of Medicine Institutional Review Board for Human Subject Research and the General Clinical Research Center Advisory Board, adolescents were recruited by local advertisement. Adolescents were screened and enrolled in the study after written assent from the participant and consent from the legal guardian were obtained. Only post-pubertal (Tanner pubertal Stages IV–V, age range 13–17 years) sedentary Hispanic adolescent girls with a normal BMI (<85th percentile/age) were included. BMI cut-off criteria were based on CDC growth charts [2]. Their sedentary behavior was defined as not participating in any school or after-school organized athletic activities and less than 45 min light to moderate physical activity per week.

All participants had parents and grandparents of Hispanic descent by self-report and reported stable body weight for at least six months. In addition, all participants were in good health as determined by medical history, a physical examination and a standard blood chemistry analysis including blood lipids, liver, as well as kidney function tests, hemoglobin, hematocrit, hemoglobin A1c and fasting and 2-h post-prandial glucose response. Furthermore, participants were not taking medications, including birth control pills, and had no first-degree relatives with diabetes. 

To evaluate the potentially specific effect of high body fat % on metabolic parameters, the adolescents (all of whom had a normal BMI of <85th percentile/age (N-BMI)) were partitioned by their body fat content: High Fat (HF) girls: body fat ≥27%; Normal Fat (NF) girls: body fat <27%. Body fat % criteria were based on data from Shypailo et al. [28].

### 2.2. Study Design

Three days prior to the study, each participant received a controlled diet with a fixed macronutrient distribution to exclude the effects of dietary intake on the experimental results (identical in all participants) as previously described [8,16]. Participants were admitted to the metabolic research unit at Children’s Nutrition Research Center Houston, Texas, the evening before the study, and weight and height measurements were obtained. Dual-energy X-ray Absorptiometry (DXA) was obtained at screening and was repeated if more than a month had passed at the time of the study.

After a 12-h overnight fast (except for water), two fasting blood samples were obtained, and a standard (1.75 g/kg bodyweight (up to 75 g)) 3-h, oral glucose tolerance test (OGTT) was performed as described by Yeckel et al. [29].

Subsequently, the participants were transferred to the radiology department at Texas Children’s Hospital for Magnetic Resonance Imaging and Spectroscopy (MRI/MRS) of abdominal and hepatic fat content, respectively.

### 2.3. Body Composition Analyses

Non-bone lean body mass, fat mass and body fat % were measured and calculated by DXA (QDR 11.2; Hologic Inc., Bedford, MA, USA) [8,16,27]. Abdominal fat content was measured by MRI and intrahepatic fat content by MRS using a Philips Achieva 1.5T whole body scanner, Software Release 1.5, (Best, Holland) [8]. The MR image of abdominal fat, i.e., visceral and subcutaneous fat content, was acquired in a single transversal slice at the level of the umbilicus. MRI data are expressed as cross-sectional area (cm^2^). Hepatic fat content is expressed as the total lipid/water peak area ratio (%) [8]. Hepatic fat was considered normal if the MRS lipid peak/water peak was <5.6% and high if the MRS lipid peak/water peak was >5.6% [8,30].

### 2.4. Biochemical Analyses

Plasma concentrations of glucose and insulin were measured to calculate insulin resistance and insulin sensitivity. Adipocyte dysfunction was evaluated by measuring plasma concentrations of leptin and adiponectin. Whole body inflammation was assessed by measuring the plasma concentration of the downstream marker of inflammation, high sensitivity C-reactive protein (Hs-CRP) [14]. All biochemical parameters were measured as previously described [16]. Hs-CRP categorizes cardiovascular disease risk in adults as follows: Hs-CRP <1.0 mg/L lowest risk; 1.0–3.0 mg/L average risk; >3.0 mg/L highest risk [31].

Insulin resistance was calculated by the Homeostasis model Assessment (HOMA-IR: fasting insulin μU/mL × fasting glucose mmol/l /22.5) [32]. HOMA-IR > 3.29 was considered insulin resistant [33]. Insulin sensitivity was calculated by the Whole Body Insulin Sensitivity Index (WBISI: (10,000/√((fasting glucose × fasting insulin)×(mean glucose 120 min × mean insulin 120 min))) [29,34]. Currently, no WBISI cut-off value exists to define insulin resistance.

### 2.5. Statistical Methods

Data are presented as the mean ± SD. Pearson correlation was used to test for correlations between variables. Differences between groups were assessed by a two-sided unpaired *t*-test. *p* ≤ 0.05 was considered statistically significant.

## 3. Results

### 3.1. Clinical Characteristics

Demographic and body composition characteristics of the girls with a N-BMI-HF (*n* = 15) and with a N-BMI-NF% (*n* = 8), as well as that of the whole group of N-BMI participants (results of N-BMI-NF and N-BMI-HF combined; N = 23) are presented in Table 1.

The subjects’ pubertal stage, age and height were similar between the two N-BMI groups. However, weight (*p* < 0.01), BMI (*p* < 0.01), body fat %, (*p* < 0.01) fat mass (*p* < 0.01) subcutaneous fat (*p* < 0.01), visceral fat (*p* < 0.05) and hepatic fat (*p* < 0.01) were all higher in the N-BMI-HF girls.

### 3.2. Metabolic Risk Indicators

Metabolic risk indicators of the N-BMI-HF (*n* = 15), the N-BMI-NF (*n* = 8), as well as that of the whole N-BMI group are presented in Table 2. The N-BMI-HF group had higher plasma insulin (*p* ≤ 0.05), leptin (*p* ≤ 0.01) and Hs-CRP (*p* ≤ 0.05) concentrations and higher HOMA-IR values (*p* ≤ 0.05), when compared to those of the N-BMI-NF adolescents. Moreover, all participants with signs of insulin resistance and a high CRP were in the N-BMI-HF group. Below BMI 22.4 kg/m^2^, no insulin resistance was observed. No difference between groups was found in adiponectin concentrations despite the fact that the mean values were lower in the HF group.

### 3.3. Correlations of N-BMI (N-BMI-NF and N-BMI-HF Combined)

**Figure 1:**
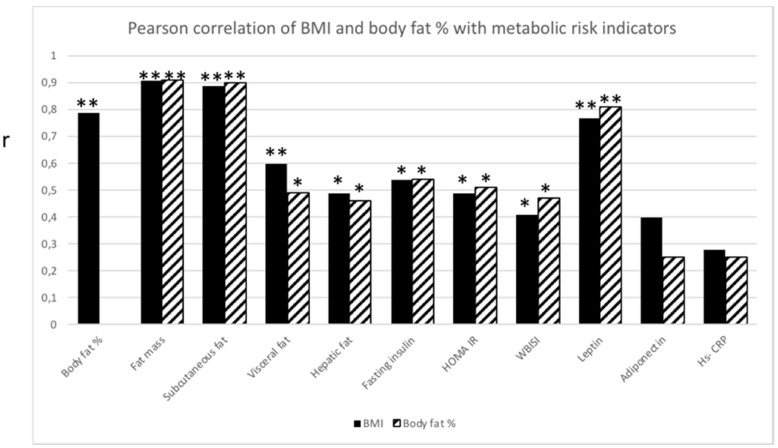
Pearson correlation analysis between BMI (black bar) and body fat % (striped bar), respectively, and metabolic risk indicators. Significant correlation (r) between BMI or body fat % with metabolic risk indicators. * *p* ≤ 0.05; ** *p* ≤ 0.01.

BMI and body fat % were significantly correlated (*p* ≤ 0.01). Not surprisingly, both BMI and body fat % correlated with fat mass (*p* ≤ 0.01), subcutaneous fat (*p* ≤ 0.01) and visceral fat (*p* ≤ 0.01 and *p* ≤ 0.05, respectively). Both BMI and body fat % correlated with, hepatic fat (*p* ≤ 0.05), fasting insulin (*p* ≤ 0.05), HOMA IR (*p* ≤ 0.05), WBISI (*p* ≤ 0.05) and leptin (*p* ≤ 0.01) (Figure 1).

**Figure 2:**
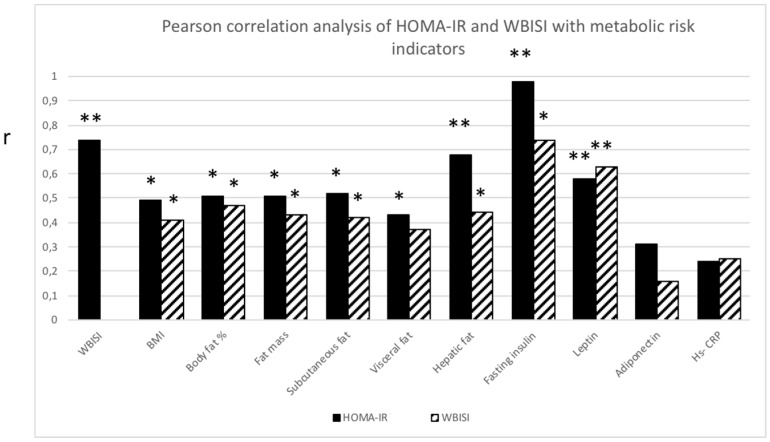
Pearson correlation analysis between homeostasis Model Assessment Insulin Resistance (HOMA-IR; black bar) and Whole Body Insulin Sensitivity Index (WBISI; striped bar) with metabolic risk indicators. Significant correlation (r) between HOMA-IR or WBISI with metabolic risk indicators. * *p* ≤ 0.05 ** *p* ≤ 0.01.

Both HOMA-IR and WBISI correlated with all measures of abdominal fat distribution (*p* ≤ 0.05) except for visceral fat, which solely correlated with HOMA-IR (*p* ≤ 0.05). In addition, both parameters correlated significantly with leptin (*p* ≤ 0.01), but not with adiponectin or Hs-CRP (Figure 2).

## 4. Discussion

This study shows that a normal BMI in sedentary Hispanic adolescent girls is not an indicator of the absence of metabolic risk. In this group of subjects, a high body fat %, also in the presence of a normal BMI, is associated with metabolic risk factors and altered body composition. These girls present increased fat mass, abdominal fat deposits, insulin resistance and increased plasma concentrations of insulin and leptin. Moreover, low grade chronic whole body inflammation, measured by high Hs-CRP, was detected in adolescents with a high body fat %, as previously described in obese adolescents [15,16]. Collectively, these data indicate that these girls with a BMI <85th percentile and a high body fat % have an increased risk of cardiovascular disease [14,35,36] as recently described in a Finish seven-year longitudinal study in children and adolescent girls with normal BMI, but high body fat % [37].

High adiponectin concentration is purported to have a protective effect on insulin sensitivity partly attributed to its anti-inflammatory function [18] and is lower in obese adolescents [38]. The lack of correlation between insulin resistance measures and adiponectin concentrations in our participants suggest that within the normal BMI range, insulin resistance and low grade whole body inflammation may not be the result of a diminished “protective effect” of this adipokine. Nonetheless, a non-significant progressive decline in adiponectin can be observed in previously studied high BMI adolescent girls (>95th percentile for age; adiponectin 6.6 ± 3.0 mg/mL) [8,16,27] as compared to N-BMI-HF and N-BMI-NF adolescents, indicating the metabolic role of this adipokine in obesity-related disease. However, since total adiponectin and not the physiologically more active High Molecular Weight (HMW) adiponectin was measured, a potential difference of HMW adiponectin distribution between groups cannot be excluded [39].

Increased abdominal fat, specifically visceral fat [40], is an important contributor to obesity-related risk indicators in obese adolescents [8,10,11,13,40,41] in part by contributing to the chronic state of whole body inflammation [40]. In addition, subcutaneous fat content has also been related to metabolic disorders in pre-pubertal children [41,42]. It has been suggested that the accumulation of subcutaneous fat may be a normal physiological response that prevents storage of fat accumulation in ectopic sites such as liver and muscle to decrease the risk of obesity-related metabolic disturbances [9,13]. The progressive increase in visceral and hepatic fat, combined with the increased insulin resistance, suggests that the subcutaneous adipose tissue is insufficient to take up fat in our studied sedentary normal BMI Hispanic adolescents (adiposopathy) [12,13]. Although hepatic fat content remained within the normal range in all participants, higher hepatic fat in HF girls warrants attention. No follow-up study could be done in the studied group of subjects to determine if these adolescents with higher hepatic fat actually developed non-alcoholic fatty liver disease [8,23,43].

The results of the present study demonstrate that a normal BMI, as currently defined, is not a good indicator of the absence of metabolic risk in this group of Hispanic adolescent girls. A high body fat % in these girls is related to increased metabolic risk indicators. Currently, no consensus exists on the definition of normal body fat % [5,28]. Here, we demonstrate that metabolic derangements are present in normal BMI adolescent girls with a body fat % ≥27, indicating that using the higher body fat % cut-off criteria published by Flegal et al. [5] (average body fat % of Mexican American girls of 34.7) results in a dramatic underestimation of metabolic risk. The problem with the current cut-off criteria using BMI or body fat % is that these criteria solely rely on the distribution within a certain population rather than on the relation between BMI or body fat % and the presence of actual metabolic alterations, as reconfirmed in obese children [44]. To identify adequately a population at metabolic risk, we need better BMI and body fat distribution cut-off criteria that would involve the ethnicity, race and sex of the individuals. This problem has particularly been recognized in the Asian ethnic groups [19,20,21] and resulted in the utilization of lower cut-off of BMI to define obesity in the Asian population [19,20]. A weakness of the present study is the small sample size. However, it is representative for the large body fat % database of normal BMI Hispanic, female adolescents developed by Dr. K. J. Ellis (Children’s Nutrition Center, Houston, TX, USA) (data not shown). Other potential limitations of the study are the evaluation of sedentary behavior by self-report and the lack of registration of tobacco use, and although the participants received a controlled diet prior to the study, the effects of prior food habits/diet were not completely ruled out.

In conclusion, sedentary, post-pubertal Hispanic, adolescent girls with a normal BMI can show a body fat %, fat distribution and metabolic profile that puts them at risk for the development of obesity-related morbidity as is known to occur in Hispanics [22,23,24,25]. The development of BMI and body fat % distribution cut-off criteria per sex, age and racial group based on metabolic risk indicators is needed in order to optimize the effectiveness of metabolic risk screening procedures, or a new and more precise measure to determine whole body adiposity needs to be created.

## Figures and Tables

**Table 1:** Clinical characteristics and body composition (mean ± SD).

	N-BMI	N-BMI-NF	N-BMI-HF
N	23	8	15
Pubertal stage (Tanner)	IV–V	IV–V	IV–V
Age (y)	14.3 ± 1.3	14.0 ± 1.1	14.5 ± 1.5
Height (m)	1.56 ± 0.1	1.55 ± 0.1	1.55 ± 0.1
Weight (kg)	50.9 ± 7.7	43.2 ± 4.4	55.1 ± 5.6 **
BMI (kg/m^2^)	20.8 ± 2.5	18.0 ± 0.9	22.3 ± 1.6 **
Body fat %	29.3 ± 5.3	23.1 ± 1.0	32.5 ± 2.6 **
Lean body mass (kg)	34.5 ± 3.9	32.0 ± 3.3	35.9 ± 3.6 **
Fat mass (kg)	15.5 ± 4.8	10.1 ± 1.6	18.4 ± 3.0 **
Subcutaneous fat (cm^2^)	184 ± 71	110 ± 37	224 ± 49 **
Visceral fat (cm^2^)	16 ± 10	10 ± 6	18 ± 10 *
Hepatic fat (%)	1.0 ± 0.7	0.5 ± 0.2	1.2 ± 0.8 **

N-BMI = mean of N-BMI-NF and N-BMI-HF combined; N-BMI-NF = Normal BMI Normal body Fat %; N-BMI-HF = Normal BMI High body Fat %. Different between N-BMI-NF and N-BMI-HF participants. * *p* ≤ 0.05 ** *p* ≤ 0.01.

**Table 2:** Metabolic risk indicators (mean ± SD).

	N-BMI	N-BMI-NF	N-BMI-HF
N	23	8	15
Glucose (mmol/L)	4.86 ± 0.37	4.91 ± 0.33	4.81 ± 0.40
Insulin (μU/mL)	10.6 ± 4.8	7.9 ± 3.0	12.0 ± 5.0 *
HOMA-IR	2.29 ± 1.1	1.73 ± 0.7	2.59 ± 1.2 *
WBISI	4.0 ± 1.7	4.9 ± 2.0	3.5 ± 1.4
Leptin (ng/mL)	20.0 ± 12.2	8.7 ± 3.5	27.5 ± 9.8 **
Adiponectin (mg/mL)	11.6 ± 6.6	14.4 ± 9.5	10.1 ± 4.0
Hs-CRP (mg/L)	0.96 ± 1.39	0.31 ± 0.19	1.30 ± 1.62 *

N-BMI = mean of N-BMI-NF and N-BMI-HF combined. Different between N-BMI-NF and N-BMI-HF participants. * *p* ≤ 0.05 ** *p* ≤ 0.01.
